# Assessment of the Financial Health of Rural Hospitals After Implementation of the Georgia Rural Hospital Tax Credit Program

**DOI:** 10.1001/jamanetworkopen.2021.17791

**Published:** 2021-07-23

**Authors:** Bettye A. Apenteng, Samuel T. Opoku, Charles Owens, Emmanuel Akowuah, Linda Kimsey, Angie Peden

**Affiliations:** 1 Department of Health Policy and Community Health, Jiann-Ping Hsu College of Public Health, Georgia Southern University, Statesboro; 2 Center for Public Health Practice and Research, Jiann-Ping Hsu College of Public Health, Georgia Southern University, Statesboro; 3 Department of Public Health, Baylor University, Waco, Texas

## Abstract

**Importance:**

In 2016, Georgia implemented the Rural Hospital Tax Credit Program, which allows taxpayers to receive a tax credit for contributions to qualifying rural hospitals in the state. Empirical evidence of the program’s association with the viability of the state’s rural hospitals is needed.

**Objective:**

To examine the association of the tax credit program with the financial health of participating rural hospitals.

**Design, Setting, and Participants:**

This longitudinal cross-sectional study used hospital financial data from the Centers for Medicare & Medicaid Services for 2015 to 2019. A difference-in-differences analytic approach was used to examine the association of the tax credit program with rural hospital financial health. Study participants included Georgia rural hospitals eligible to participate in the program. Comparison hospitals were selected from the southern states of Alabama, Florida, Mississippi, North Carolina, South Carolina, and Tennessee.

**Exposures:**

Hospital participation in the Georgia Rural Hospital Tax Credit Program.

**Main Outcomes and Measures:**

The primary outcome of the study was financial health measured with total margin, days cash on hand, debt-asset ratio, and average age of plant as well as a Financial Strength Index (FSI), which combined the previous measures to assess overall financial strength.

**Results:**

The analytical sample included a balanced panel of 136 hospitals, with 47 Georgia Rural Hospital Tax Credit Program participants (18 [38%] critical access hospitals; 5 [11%] system affiliated; mean [SD] bed count, 60 [47]; mean [SD] Medicare inpatient mix, 52% [16]) and 89 comparison hospitals (43 [48%] critical access hospitals; 24 [27%] system affiliated; mean [SD] bed count, 52 [41]; mean [SD] Medicare inpatient mix, 67% [18]). Two years after implementation, program participation was associated with a 23% increased probability of good or excellent financial health (*b* = 0.23; 95% CI, 0.10-0.37; *P* < .001) and a 6.7-point increase in total margin (*b* = 6.67; 95% CI, 3.61-9.73; *P* < .001).

**Conclusions and Relevance:**

These early findings suggest that the Georgia Rural Hospital Tax Credit Program is associated with improvements in hospital financial health; however, additional studies are needed to assess the program’s long-term impact on the financial sustainability of Georgia’s rural hospitals.

## Key Points

**Question:**

Is the Georgia Rural Hospital Tax Credit Program associated with the financial health of participating rural hospitals 2 years after implementation?

**Findings:**

In this cross-sectional study of 136 hospitals, difference-in-differences analysis found that the tax credit program was associated with increases in program participants’ probability of reporting good or excellent financial strength, which appeared to be driven by an increase in profitability.

**Meaning:**

These findings suggest that the Georgia Rural Hospital Tax Credit program has improved the financial health of participating rural hospitals.

## Teaser

This cross-sectional study examines the association of Georgia’s Rural Hospital Tax Credit Program with the financial health of participating rural hospitals.

## Introduction

In 2016, the state of Georgia passed Senate Bill (SB) 258, which created the Rural Hospital Tax Credit Program. The program, which went into effect on January 1, 2017, allows taxpayers filing as individuals, married, or as corporations to receive a tax credit for contributions to a qualifying rural hospital in the state of Georgia. Single individuals and those married and filing jointly can claim 100% of donated funds up to $5000 and $10 000, respectively. Corporations can claim the lesser of 100% of contributions or 75% of state liabilities. Statewide contributions to the program are capped annually; the cap for 2021 is $60 million.^1^ The donations received by participating rural hospitals can be allocated for regular operational expenses, debt payment, capital improvements, equipment, and other investments.^2^

The Rural Hospital Tax Credit Program’s aim is to improve the financial sustainability of the state’s rural hospitals. This is a critical aim, given that 9 of Georgia’s rural hospitals have closed since 2008.^3^ In addition, other studies suggest that many other rural hospitals in the state and across the nation are struggling financially, and several are leaning toward closure.^4^ The closure of hospitals has the potential to destabilize an already fragile rural health care infrastructure. Hospital closures can adversely impact health care access^5^ and may even increase inpatient mortality in affected rural communities.^6^

Analysis of a program expenditure report provided by the Georgia Department of Community Health indicates that in 2018, approximately $59.5 million in total donations was received under the Rural Hospital Tax Credit Program, with eligible hospitals receiving, on average, approximately $1 million each.^7^ While fund use varied by hospital, eligible hospitals used funds primarily to supplement regular hospital operations, for capital expenses, technology purchases, and debt repayment.^7^

Recent evidence suggests that Georgia’s Rural Hospital Tax Credit Program may help struggling rural hospitals in the state. A previous qualitative study of the program^8^ reported favorable perceptions about the program among Georgia rural hospital executives, who described the program as an important lifeline, especially for severely struggling hospitals. They reported investing donations from the program directly into hospital operations and growth efforts, including servicing debt, supporting service expansion efforts, and investing in technology and capital infrastructure.^8^ While this qualitative study represented an initial attempt to assess how the tax credit program has affected rural hospital viability, empirical evidence is needed. As we advance discussions on how to improve the financial health of rural hospitals, especially after the potentially adverse financial impact of the COVID-19 pandemic,^9^ evaluations of state programs geared toward building sustainable rural health infrastructure, such as this, may add valuable insights to the ongoing dialogue. Thus, the purpose of this study was to examine the association between participation in the Georgia Rural Hospital Tax Credit Program and the financial health of rural hospitals.

## Methods

### Study Design, Setting, and Participants

The study was a longitudinal cross-sectional study assessing the association between participation in the Georgia Rural Hospital Tax Credit Program and the financial health of participating rural hospitals. Per the Common Rule, because this study did not involve human participants, institutional review board approval and informed consent were not required. We followed the Strengthening the Reporting of Observational Studies in Epidemiology (STROBE) reporting guideline.

The study sample included Georgia hospitals that were eligible to receive donations under the Georgia Rural Hospital Tax Credit Program. Comparison hospitals were selected from 6 states in the US Department of Health and Human Services (HHS) Region B, of which Georgia is a part, ie Alabama, Florida, Mississippi, North Carolina, Tennessee, and South Carolina. All states are southern states with similar hospital market factors. This regional classification is also used by the Federal Office of Rural Health and the Health Resources and Services Administration Hospital Services in their programming. HHS Region B also includes Kentucky. However, Kentucky expanded Medicaid, while the others did not. Thus, to ensure similarity in hospital market experience, Kentucky hospitals were excluded from the study sample.

Comparison hospitals were selected based on tax credit program eligibility criteria, including hospital type (ie, short-term acute hospital or critical access hospital), ownership (ie, tax exempt status or hospital authority), Medicare and Medicaid program participation, and population size (ie, 50 000 residents or fewer in the county of location). Hospitals reporting no inpatient stays, those with a cost reporting period of less than 364 days, and those without data on the outcome variables were also excluded.

Notably, in the first year of the program (2017), the state set the population eligibility criterion at 35 000 residents or fewer but expanded this to 50 000 or fewer in subsequent program years. As a result, 9 Georgia rural hospitals did not take part in the program in its inception year, which had 49 participating hospitals. These 9 hospitals subsequently participated in 2018 and 2019,^10^ bringing the total program participants to 58 rural hospitals. Furthermore, even though the program went into effect in 2017, some hospitals may not have received donations until 2018, resulting in a potential 1-year lag in financial reporting. Thus, to minimize potential bias in the estimation, we excluded data from 2017 in the difference-in-differences (DID) estimation.

After applying the inclusion and exclusion criteria, the sample included an unbalanced panel of 862 hospital-year observations. Considering the evidence that suggests that parameter estimates may be inconsistent in the presence of time varying nonresponse,^11^ the analysis was conducted with a balanced panel of 680 hospital-years that was restricted to only hospitals with complete 5-year data (2015-2019). The final analytical sample included 47 hospitals from Georgia (tax credit participants) and 89 from other states (comparison hospitals). Because we excluded 2017 from the DID estimations, the DID estimations are conducted on a balanced sample of 544 hospital-years. We used the full study sample (2017 included) in the inspection of trends.

### Data Sources

Data for the study were obtained by linking hospital organizational, financial, and market level data from 3 sources: the Centers for Medicare & Medicaid Services (CMS) Provider of Service Files (for hospital organizational characteristics), the CMS Hospital Cost Reports (for hospital financial information), and the University of Wisconsin County Health Rankings (for market level characteristics). Data were obtained for the years 2015 to 2019 for each data source.

### Measures

#### Outcome Variables

The primary outcome variables assessed included profitability (measured with total margin), liquidity (measured with days cash on hand), capital structure (measured with debt-asset ratio), and the financial age of fixed assets (measured with average age of plant). While the potential impact of donations on total margin and days cash on hand is more apparent, program participation could also potentially be associated with decreases in debt-asset ratio and average age of plant, as donations received by participating rural hospitals can be allocated for debt payment, capital improvement, and equipment purchases

Total margin was computed as the ratio of net income to total income, expressed as a percentage. Days cash on hand was computed as the ratio of the sum of cash, unrestricted investments, and marketable securities to total expenses (excluding depreciation) and standardized by the days in cost-reporting period. Hospitals reporting negative days cash on hand were excluded due to potential data reporting errors.^12^ Debt financing was computed as the ratio of the sum of total liabilities to total assets, expressed as a percentage. Finally, average age of plant was computed as follows:

accumulated depreciation / (depreciation expense × [365 / days in cost-reporting period]).

All 4 indicators were combined into a composite measure of hospital financial health: the Cleverley Financial Strength Index (FSI),^13^ which provides a more holistic picture of organizational financial health than individual financial indicators.^13,14^ It is computed as follows:

([total margin − 4.0] / 4.0) + ([days cash on hand − 50] / 50) + ([50 − debt financing percentage] / 50) + ([9.0 - average age of plant] / 9.0)

The denominators were based on industry standards at the time of the index’s development and are used to standardize each indicator.

The FSI is typically categorized as follows: poor financial strength (less than −2), fair financial strength (–2 to 0), good financial strength (0 to 3), and excellent financial strength (greater than 3).^14^ For ease of interpretation, we recoded this as a binary indicator (1, good or excellent; 0, poor or fair).

To minimize the effects of extreme outliers, financial indicators were winsorized at the 2.5th and 97.5th percentiles. Winsorization, commonly used with administrative financial data, minimizes the effect of outliers while preserving data.^15^

#### First-Order Associations

Before assessing the association of the Georgia Hospital Tax Credit Program with hospital financial outcomes, we examined its association with charitable contributions or donations to hospitals. While it is undocumented how hospitals report information on tax credits on the hospital cost reports from an accounting perspective, it seems most likely that they would be reported under the Statement of Revenues and Expenses (Worksheet G-3) as income from contributions, donations, bequests, and other similar sources. Thus, we measured donations using this data. Data for all financial indicators assessed in this study were obtained for fiscal years 2015 to 2019 for each hospital.

#### Independent Variables

We sought to explore the association of the Georgia Hospital Tax Credit Program with hospital financial performance using a DID approach. Thus, the key independent variables included variables assessing program participation (ie, Georgia program participants vs comparison hospitals); period (ie, preprogram vs postprogram implementation); and the DID indicator, the interaction between these 2 variables (ie, program participation × period). The pre-implementation period included 2015 to 2016, while the postimplementation period included 2018 and 2019.

#### Covariates

We adjusted for possible organizational- and market-level confounding factors, including type of hospital (critical access hospital vs other short-term acute hospitals), bed size, system affiliation, Medicare inpatient payer mix, the number of hospitals within the county of location as well as the county of location’s population size, proportion of elderly residents, uninsured rate, median household income, and proportion of non-Hispanic White residents. We also adjusted for the scope of services provided by the hospital. Service scope was measured as a count of the number of services offered directly by the hospital or through partnerships from a list of 31 potential services documented in the CMS Provider of Service Files. Hospital market factors are assessed at the county level in this study.

#### Georgia Rural Hospital Stabilization Program

Prior to the implementation of the Rural Hospital Tax Credit Program, Georgia implemented a grant program to stabilize the state's rural health infrastructure by improving access to timely, quality care in appropriate settings. Between 2016, when the first cohort of hospitals was enrolled in the program, and 2019, 22 rural hospitals have participated in the program. Eligibility for participation in the program is determined annually, and hospitals only participate in the program for 1 year. Each year, participating hospitals shared a total of $3 million in grants geared toward programmatic interventions to improve access to care. Participating hospitals have used grant funding from this program to expand access to primary care and to reduce readmissions and inappropriate emergency department utilization, among other initiatives.^16^ To adjust for the potential confounding effect of the Rural Hospital Stabilization Program on hospital financial health, we included a dummy variable to capture hospital participation in the hospital stabilization program for each year.

### Statistical Analysis

Adopting a DID analytical approach, we conducted multiple generalized linear models (GLMs) to assess the association of the Rural Hospital Tax Credit Program with hospital financial health, using the FSI and each of its component indicators (ie, total margin, days cash on hand, debt-asset ratio, and average plant age) as outcome measures. For normally distributed outcomes (total margin), we used a GLM modeled on a gaussian distribution with an identity link. To account for the nonnormal distribution of the days cash on hand, debt-asset ratio, and average age of plant variables, we use GLM modeled on a gaussian distribution with a log link. For ease of interpretation, the binary FSI indicator was modeled using a linear probability model (GLM on gaussian distribution with an identity link). The contribution variable used to examine the first-order associations was modeled with GLM (γ distribution, log link).

For each outcome variable, the DID parallel trends assumption was tested by interacting the year variables with the program participation variable for the pre-implementation period. A significant interaction would indicate the violation of the parallel trends assumption. We also plotted trend graphs to visually assess trends in the outcome variables for the program participants and the comparison hospitals.

Each DID model adjusted for the control variables, as previously described. Models also included year and state fixed effects, with robust standard errors clustered at the county level. Statistical significance was assessed at the 2-tailed *P* < .05 level. All analyses were completed in Stata version 16 (StataCorp).

#### Robustness Check

As a robustness check, we repeated the DID estimation with a seemingly unrelated outcome variable. We chose patient deductions as the outcome variable for the robustness check. Patient deductions were measured as the ratio of contractual allowances and discounts to gross total patient revenue, expressed as a percentage. This outcome variable was chosen for the robustness test because it is not expected that the tax credit program would be associated with contractual negotiations with payers, especially in the short term. The variable was winsorized at the 2.5th and 97.5th percentiles to minimize the effect of outliers and modeled using GLM (gaussian distribution, log link).

#### Sensitivity Analysis

To account for potential violation of the parallel trends assumption, we also conducted propensity score–matched DID estimations as sensitivity analyses. To address potential violations of the parallel trends assumption, hospitals were matched based on preperiod average values of all outcome variables.^17^ Matching was accomplished using propensity score radius matching with replacement and a caliper of 0.005.^18^ Matching resulted in a matched analytical sample of 32 tax credit participants and 58 comparison hospitals.

To rule out a potential confounding effect of the Rural Hospital Stabilization program, we repeated the DID estimations on a sample that excluded hospitals that participated in the Rural Hospital Stabilization program at any time. We also repeated the DID estimations for contributions and donations using a 2-part model (probit for first stage and GLM, γ distribution and log link, for second stage) to account for excessive zeros in this variable.

## Results

### Sample Characteristics

The analytical sample included 136 hospitals, with 47 Georgia Rural Hospital Tax Credit Program participants (18 [38%] critical access hospitals; mean [SD] bed count, 60 [47]) and 89 comparison hospitals (43 [48%] critical access hospitals; mean [SD] bed count, 52 [41]). We compared the average baseline (2015-2016) hospital organizational and market characteristics between program participants and comparison hospitals. Table 1 provides descriptive information about the organizational, market, and financial health characteristics of the study sample during the baseline period. While program participants and comparison hospitals were comparable on most organizational characteristics and market characteristics, compared with comparison hospitals, tax credit program participants were less likely to be affiliated with a system (5 [11%] vs 24 [27%]; *P* = .03) and reported lower mean (SD) Medicare inpatient payer mix (52% [16] vs 67% [18]; *P* < .001). They were also, on average, located in counties with higher mean (SD) rates of uninsured patients (21% [2] vs 19% [3]; *P* < .001) and lower mean (SD) numbers of other hospitals (1.0 [0.2] vs 1.2 [0.4]; *P* = .03). With respect to the assessed financial health indicators, the FSI and its component indicators were statistically similar between the groups in the pre-implementation period, except for average age of plant. Georgia hospitals had significantly older facilities compared with the comparison hospitals (mean [SD], 42 [87] years vs 12 [10] years; *P* < .001) (Table 1).

**Table 1.** Sample Characteristics

Variable	Unmatched sample			Matched sample^a^		
	Tax credit hospitals (n = 47)	Comparison hospitals (n = 89)	*P* value	Tax credit hospitals (n = 32)	Comparison hospitals (n = 58)	*P* value
**Hospital-level factors, mean (SD)**						
CAH, No. (%)	18 (38.3)	43 (48.3)	.27	15 (46.9)	26 (44.8)	.85
System affiliated, No. (%)	5 (10.6)	24 (27.0)	.03	3 (9.4)	25.9 (15)	.07
Bed count, No.	60.0 (47.3)	52.1 (40.9)	.32	61.0 (54.1)	52.9 (36.2)	.40
Service count, No.	14.4 (4.1)	14.7 (4.1)	.66	14.2 (4.2)	14.7 (4.4)	.65
Medicare inpatient mix, %	51.5 (15.7)	67.2 (17.9)	<.001	53.2 (16.2)	67.6 (17.1)	<.001
**Market-level factors, mean (SD)**						
Hospitals in county, No.	1.0 (0.15)	1.2 (0.42)	.03	1.0 (0.18)	1.1 (0.33)	.10
Population	21 918 (10 948)	24 119 (11 311)	.28	22 366 (11 824)	24 554 (11 059)	.38
Residents ≥65 y, %	17.4 (4.0)	17.6 (2.9)	.65	17.5 (4.4)	17.4 (2.4)	.82
Non-Hispanic White residents, %	63.8 (14.0)	63.7 (21.4)	.98	65.3 (14.4)	62.5 (20.5)	.51
Uninsured rate, %	20.9 (2.2)	18.8 (3.2)	<.001	21.1 (2.5)	18.6 (3.5)	<.001
Median household income, $	37 347 (5369)	36 164 (5230)	.22	37 759 (5265)	36 194 (4902)	.16
**Financial indicators, mean (SD)**^b^						
Financial Strength Index	–1.87 (5.7)	–0.52 (3.7)	.10	–0.57 (4.4)	–0.47 (3.2)	.91
Hospitals with good or excellent financial strength, No.	30.9 (0.45)	41.0 (0.44)	.20	39.1 (0.47)	38.8 (0.43)	.98
Total margin, %	–1.97 (7.9)	0.24 (7.8)	.12	0.09 (7.5)	0.01 (7.3)	.95
Days cash on hand	99.5 (155.3)	92.2 (101.8)	.74	92.1 (135.5)	89.0 (101.4)	.90
Debt to asset ratio, %	60.6 (60.6)	55.2 (40.2)	.49	48.9 (37.9)	56.1 (42.4)	.43
Average age of plant, y	41.6 (86.8)	12.0 (9.8)	.002	13.0 (7.3)	11.0 (8.4)	.29
Patient deduction, %	58.9 (12.5)	55.4 (14.1)	.16	56.1 913.1)	55.9 (13.5)	.95
Contributions and donations, $	195 840 (308 212)	125 566 (280 421)	.18	138 478 (270 350)	79 869 (191 223)	.24

Abbreviation: CAH, critical access hospital.

^a^ Propensity score radius matching with 0.005 caliper.

^b^ Unadjusted mean value over baseline period.

#### Parallel Trends Assumption

**Figure 1.**
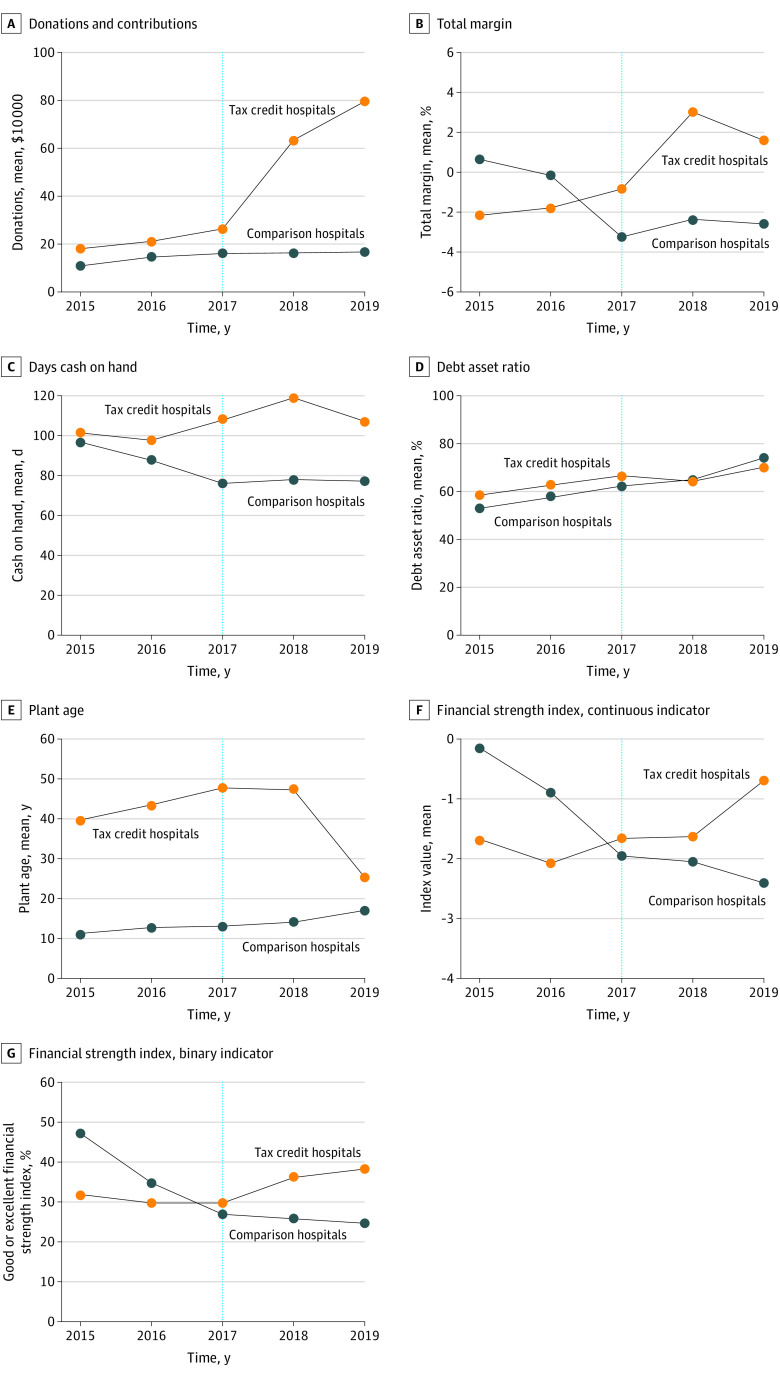
Unadjusted Linear Trends of Outcome Variables

A visual examination of the trend graphs suggested potential nonviolation of the parallel trends assumption for all outcomes (Figure 1), perhaps with the exception of the FSI binary indicator. However, an assessment of the coefficients of the linear time trend interacted with the program participation variable during the pre-implementation period showed the lack of statistical significance for all assessed indicators (eTable 1 in the Supplement). We proceeded with traditional DID estimation with an assumption of parallel trends. However, as a conservative approach, we also conducted propensity score–matched DID estimations as an additional sensitivity analyses.

### Main DID Estimation**s**

#### First-Order Association

Results from the DID estimation, adjusting for potential hospital and market characteristics, indicate a statistically significant association of Georgia Rural Hospital Tax Credit Program participation with donations received by hospitals. The DID estimator was statistically significant (exponentiation [exp] [*b*] = 7.93; 95% CI, 2.92-21.5; *P* < .001) (Table 2).

**Table 2.** Difference-in-Differences Estimation, Full Sample of 544 Hospital-Years

Model^a^	Contribution^b^	Total margin^c^	Days cash on hand^d^	Debt-asset ratio^d^	Average plant age^d^	FSI^c^
	exp (*b*) (SE) [95% CI)	*b *(SE) [95% CI)	exp (*b*) (SE) [95% CI)	exp (*b*) (SE) [95% CI)	exp (*b*) (SE) [95% CI)	*b *(SE) [95% CI)
Program participation vs not participating	0.42 (0.32) [0.09 to 1.87]	–0.10 (2.01) [–4.04 to 3.85]	2.41 (1.69) [0.61-9.52]	1.12 (0.27) [0.70 to 1.80]	269.08 (449.06) [10.22 to 7086.75]^e^	0.03 (0.11) [–0.20 to 0.25]
Postimplementation vs pre-implementation	1.03 (0.42) [0.46 to 2.27]	–3.89 (1.36) [–6.55 to 1.22]^e^	0.84 (0.27) [0.46 to 1.57]	1.35 (0.21) [1.00 to 1.82]	0.02 (0.02) [0.001 to 0.23]^e^	–0.20 (0.07) [–0.34 to –0.05]^e^
Program participation × time	7.93 (4.04) [2.92 to 21.45]^f^	6.67 (1.56) [3.61 to 9.73]^f^	1.18 (0.20) [0.85 to 1.64]	0.96 (0.13) [0.73 to 1.26]	0.76 (0.67) [0.13 to 4.30]	0.23 (0.07) [0.10 to 0.37]^f^

Abbreviations: exp (*b*), exponentiation of *b*; FSI, Financial Strength Index.

^a^ Clustered robust SEs obtained. Models were adjusted for all hospital and market covariates listed in Table 1 and included state and year fixed effects.

^b^ Generalized linear model with γ distribution and log link.

^c^ Generalized linear model with normal distribution and identity link.

^d^ Generalized linear model with normal distribution and log link.

^e^ *P* < .01.

^f^ *P* < .001.

#### Association With Hospital Financial Health

**Figure 2.**
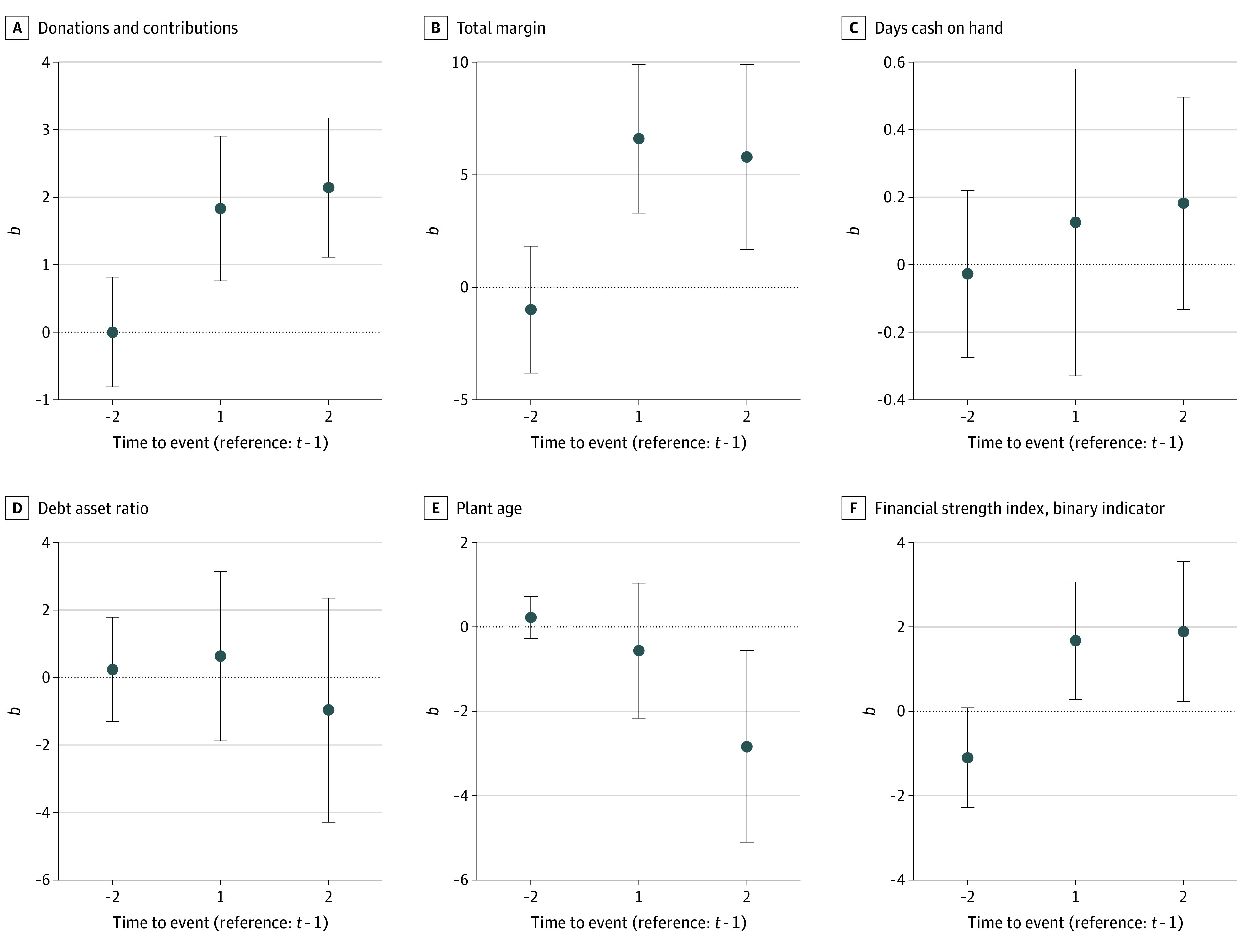
Event Study Graphs

Participation in the Georgia Rural Hospital Tax Credit Program was associated with increased financial strength, which appeared to be driven by an increase in total margin. Specifically, a statistically significant DID estimator was observed for the FSI binary indicator (*b* = 0.23; 95% CI, 0.10-0.37; *P* < .001), indicating 23% increased probability of good or excellent financial health, and total margin (*b* = 6.67; 95% CI, 3.61-9.73; *P* < .001) but not for the other financial indicators (Table 3). DID estimates are plotted as event study graphs in Figure 2 (eg, contributions and donations, postperiod year 1: *b* = 1.93; 95% CI, 0.82-3.03; *P* < .01; contributions and donations, postperiod year 2: *b* = 2.24; 95% CI, 1.18-3.31; *P* < .001; total margin, postperiod year 1: *b* = 6.60; 95% CI, 3.30-9.91; *P* < .001; total margin, postperiod year 2: *b* = 5.78; 95% CI, 1.66-9.90; *P* < 01).

**Table 3.** Difference-in-Differences Estimation, Matched Sample of 360 Hospital-Years

Model^a^	Contribution^b^	Total margin^c^	Days cash on hand^d^	Debt-asset ratio^d^	Average plant age^d^	FSI^c^
	exp (*b*) (SE) [95% CI)	*b* (SE) [95% CI)	exp (*b*) (SE) [95% CI)	exp (*b*) (SE) [95% CI)	exp (*b*) (SE) [95% CI)	*b* (SE) [95% CI)
Program participation vs not participating	0.26 (0.19) [0.06 to 1.13]	3.51 (2.34) [–1.07 to 8.09]	2.25 (1.85) [0.45 to 11.29]	0.95 (0.58) [0.29 to 3.13]	1.54 (0.78) [0.57 to 4.14]	0.22 (0.15) [–0.06 to –0.51]
Postimplementation vs pre-implementation	0.75 (0.38) [0.28 to 2.03]	–3.60 (1.28) [–6.11 to –1.09]^e^	0.78 (0.25) [0.42 to 1.45]	1.22 (0.64) [0.44 to 3.39]	1.45 (0.54) [0.70 to 3.00]	–0.15 (0.09) [–0.33 to 0.03]
Program participation × time	9.60 (5.32) [3.24 to 28.43]^f^	5.56 (1.72) [2.20 to 8.92]^e^	1.42 (0.31) [0.92 to 2.17]	0.86 (0.20) [0.54 to 1.36]	0.82 (0.11) [0.63 to 1.05]	0.17 (0.09) [–0.006 to 0.35]^g^

Abbreviations: exp (*b*), exponentiation of *b*; FSI, Financial Strength Index.

^a^ Clustered robust SEs obtained. Models were adjusted for all hospital and market covariates listed in Table 1 and included state and year fixed effects.

^b^ Generalized linear model with γ distribution and log link.

^c^ Generalized linear model with normal distribution and identity link.

^d^ Generalized linear model with normal distribution and log link.

^e^ *P* < .01.

^f^ *P* < .001.

^g^ *P* = .06.

### Sensitivity Analyses and Robustness Check

#### Propensity Score Matching

Matching balanced the treatment and comparison groups in terms of baseline outcomes (Table 1; eFigure 1 in the Supplement). Like the unmatched sample, tax credit and comparison hospitals were comparable with respect to most market and organizational characteristics, except for Medicare inpatient payer mix and county uninsured rate (Table 1). A formal test of the parallel trends assumption supported the use of DID estimation (eTable 2 in the Supplement). Results from the propensity score–matched DID are presented in Table 3 and were generally consistent with findings from the main estimations. However, the DID parameter estimates from the matched model were slightly smaller for the total margin (*b* = 5.56; 95% CI, 2.20 to 8.92; *P* = .001) and FSI indicators (*b* = 0.17; 95% CI, –0.006 to 0.35; *P* = .06) but larger for the donation indicator (exp [*b*] = 9.60; 95% CI, 3.24 to 28.43; *P* < .001). Of note, the DID estimate FSI binary indicator was no longer significant at the *P* < .05 level (*P* = .06) (Table 3).

#### Falsification Test

As expected, the DID estimator for patient deduction was not statistically significant (exp [*b*] = 0.99; 95% CI, 0.96-1.02; *P* = .54). Results were consistent when estimation was repeated with the propensity matched sample (exp [*b*] = 1.00; 95% CI, 0.95-1.04; *P* = .926) (eTable 3, eFigure 2, and eFigure 3 in the Supplement).

#### Additional Models

Conditioned on having nonzero donations, the tax credit programs remained statistically associated with donations and contributions in unmatched (exp [*b*] = 3.63; 95% CI, 1.97-6.62; *P* < .001) and matched (exp [*b*] = 7.46; 95% CI, 3.86-14.44; *P* < .001) analyses. However, the effect sizes were comparatively smaller (eTable 4 in the Supplement). The findings from analyses excluding Hospital Stabilization Program participants (20 hospitals) were also generally consistent with those of the main models (eTable 5 and eTable 6 in the Supplement).

## Discussion

The purpose of this study was to examine the association of the Georgia Rural Hospital Tax Credit Program with hospital financial health. The findings suggest that the program was associated with improved financial strength, which appeared to be primarily driven by an increase in total margin. More time may be needed to observe an association with indicators of liquidity, capital structure, and age of fixed assets. Notably, an examination of trends (Figure 1E and Figure 2E) suggested that the tax credit program may have a potentially lagged association with the average age of plant.

While these early findings demonstrate the promise of the Georgia Rural Hospital Tax Credit Program, additional studies are needed to assess the long-term impact of the program on rural hospital sustainability. For example, in addition to directly providing financial support for rural hospitals, rural hospital executives have expressed hope that the tax credit program will engender a sense of ownership among rural donors, resulting in increased community engagement, reduced outmigration, and increased operational profitability for rural hospitals.^8^ Future studies may be needed to comprehensively assess the impact of the program on rural bypass behavior and hospital operational profitability.

Additionally, given that the success of this program may be pivoted on taxpayer donations, future studies may be warranted to assess the sustainability of the program given recent changes in federal tax laws. The ability to claim both state tax credit and federal charitable deductions (what is known as double dipping) made the Rural Hospital Tax Credit Program attractive to taxpayers, especially high-income earners who faced an alternative minimum tax (AMT).^19^ However, the 2017 Tax Cuts and Jobs Act increased the standard deduction, which significantly reduced the number of taxpayers who itemized their federal deduction as well as those who faced AMT. With the elimination of itemization, charitable donations become less beneficial for some taxpayers. A previous study of Georgia rural hospital executives^8^ noted decreases in donations in program (calendar) year 2019 compared with 2018 levels as the federal tax credit reform went into effect.^8^ Donations decreased by approximately $10 million ($59.5 million in 2018 vs $49.4 million in 2019).^20^ Because of the misalignment between the Rural Hospital Tax Credit Program year and most hospitals’ fiscal years, these declines will most likely be reflected in fiscal year 2020 hospital cost-report data. If decreases in donations to the program continue, the financial benefits of the program may wane as well.

Georgia’s unique tax credit approach to stabilizing rural hospitals is one of several state approaches to improve rural health care systems. For example, Pennsylvania recently implemented a multipayer global budget model for rural hospitals,^21^ while Texas increased Medicaid inpatient rates for rural hospitals.^22^ However, despite these ongoing efforts, evaluations of the impact as well as comparative effectiveness of these approaches are generally lacking. As a strength, this study represents an early attempt to assess contemporary approaches by states to improve the financial viability of rural hospitals

### Limitations

There are limitations of this study. First, the study depended on administrative data sets for hospital operational and financial information that may have contained errors. To minimize the effect of data reporting errors, we winsorized the outcome variables used in the study to minimize the effect of data outliers, as commonly done for hospital financial data.^15^ Second, while the Rural Hospital Tax Credit program operates on a calendar year, hospitals operate and report their cost report information to CMS on a fiscal year basis that may not necessarily align with the calendar year. This misalignment may have biased our estimates.

Additionally, while the DID estimation seeks to obtain causal estimates on the effect of a policy or program, the approach is quasi-experimental and cannot definitively prove causality. The approach also assumes that the treatment and comparison groups are exposed to the same temporal trends and market factors, which may not have been the case. It is possible that any observed positive association with financial health may be due to factors, or a combination of factors specific to the Georgia market, other than the tax credit program. To minimize potential biases, the models included state dummy variables to account for state-level heterogeneity among the comparison states. Another concern was the potential confounding effect of the Georgia Rural Hospital Stabilization Program. However, a sensitivity analysis excluding Rural Hospital Stabilization participants generally yielded results consistent with the main finding. Furthermore, the DID estimation process is pivoted on the parallel trends assumption, which can be difficult to prove. To address any potential violation of the parallel trends assumption with our data, we estimated alternative DID models using a propensity matched sample, and the findings were generally consistent.

## Conclusions

This study found that the Georgia Rural Hospital Tax Credit Program was associated with improvements in the profitability of participating hospitals. However, with recent changes in tax laws that may negatively affect the volume of future donations to the program, more research is needed on the program’s longer-term sustainability.

## Notes

Supplement. **eTable 1. **Tests of Parallel Trends Assumption: Unmatched Sample (N = 272) **eTable 2. **Tests of Parallel Trends Assumption: Matched Sample (n = 180) **eTable 3. **Falsification Test: Patient Deduction (N = 544) **eTable 4. **Two Part DID Model: Donations and Contributions **eTable 5. **Unmatched DID Estimation: Sample Excluding Hospital Stabilization Program Participants (n = 464) **eTable 6. **Matched DID Estimation: Sample Excluding Hospital Stabilization Program Participants (n = 312) **eFigure 1. **Bias Reduction Following Matching **eFigure 2.** Unadjusted Linear Trends in Patient Deductions **eFigure 3. **Event Study Graphs of DID Estimates: Patient Deductions
