# Supplementary material for: Assessment of the Financial Health of Rural Hospitals After Implementation of the Georgia Rural Hospital Tax Credit Program
Source: JAMA Netw Open. 2021 Jul 23;4(7):e2117791. doi: 10.1001/jamanetworkopen.2021.17791 (PMC8303099; doi:10.1001/jamanetworkopen.2021.17791)
Supplement: Supplement. — eTable 1. Tests of Parallel Trends Assumption: Unmatched Sample (N = 272) eTable 2. Tests of Parallel Trends Assumption: Matched Sample (n = 180) eTable 3. Falsification Test: Patient Deduction (N = 544) eTable 4. Two Part DID Model: Donations and Contributions eTable 5. Unmatched DID Estimation: Sample Excluding Hospital Stabilization Program Participants (n = 464) eTable 6. Matched DID Estimation: Sample Excluding Hospital Stabilization Program Participants (n = 312) eFigure 1. Bias Reduction Following Matching eFigure 2. Unadjusted Linear Trends in Patient Deductions eFigure 3. Event Study Graphs of DID Estimates: Patient Deductions [file jamanetwopen-e2117791-s001.pdf]

Supplemental Online Content

Apenteng BA, Opoku ST, Owens C, Akowuah E, Kimsey L, Peden A. Assessment of the financial health of rural hospitals after implementation of the Georgia Rural Hospital Tax Credit Program. *JAMA Netw Open.* 2021;4(7):e2117791. doi:10.1001/jamanetworkopen.2021.17791

eTable 1. Tests of Parallel Trends Assumption: Unmatched Sample (N = 272)

eTable 2. Tests of Parallel Trends Assumption: Matched Sample (n = 180)

eTable 3. Falsification Test: Patient Deduction (N = 544)

eTable 4. Two Part DID Model: Donations and Contributions

eTable 5. Unmatched DID Estimation: Sample Excluding Hospital Stabilization Program Participants (n = 464)

eTable 6. Matched DID Estimation: Sample Excluding Hospital Stabilization Program Participants (n = 312)

eFigure 1. Bias Reduction Following Matching

eFigure 2. Unadjusted Linear Trends in Patient Deductions

eFigure 3. Event Study Graphs of DID Estimates: Patient Deductions

This supplemental material has been provided by the authors to give readers additional information about their work.

© 2021 Apenteng BA et al. *JAMA Network Open.*

eTable 1. Tests of Parallel Trends Assumption: Unmatched Sample (N = 272)

	Donations[a]	Total Margin[b]	Days Cash on Hand[c]	Debt Asset[c]	Average Plant Age[c]	FSI[b]	Patient Deductions[c]
	exp(b)<br>(S.E)<br>[95% CI]	*b*<br>(S.E)<br>[95% CI]	*exp(b)*<br>(S.E)<br>[95% CI]	*exp(b)*<br>(S.E)<br>[95% CI]	*exp(b)*<br>(S.E)<br>[95% CI]	*b*<br>(S.E)<br>[95% CI]	*exp(b)*<br>(S.E)<br>[95% CI]
Program Participation * 2016 (Ref: Program Participation * 2015)	0.88<br>(0.30)<br>[0.45 – 1.71]	1.18<br>(1.42)<br>[-1.60 – 3.96]	1.06<br>(0.06)<br>[0.95 – 1.20]	0.99<br>(0.07)<br>[0.85 – 1.15]	0.97<br>(0.05)<br>[0.88 – 1.06]	0.10<br>(0.06)<br>[-0.01 – 0.22]	0.99<br>(0.01)<br>[0.97 – 1.02]

[a]GLM, gamma distribution and log link. [b]GLM, normal distribution, identity link. [c]GLM, normal distribution, log link. Clustered robust standard. Analysis restricted to the pre-treatment period (N=272)

© 2021 Apenteng BA et al. *JAMA Network Open.*

eTable 2. Tests of Parallel Trends Assumption: Matched Sample (n = 180)

	Donations[a]	Total Margin[b]	Days Cash on Hand[c]	Debt Asset[c]	Average Plant Age[c]	FSI[b]	Patient Deductions[c]
	exp(b) (S.E) [95% CI]	*b* (S.E) [95% CI]	*exp(b)* (S.E) [95% CI]	*exp(b)* (S.E) [95% CI]	*exp(b)* (S.E) [95% CI]	*b* (S.E) [95% CI]	*exp(b)* (S.E) [95% CI]
Program Participation * 2016 (Ref: Program Participation * 2015)	1.48 (0.51) [0.75 – 2.90]	0.63 (1.70) [-2.70– 3.97]	1.06 (0.08) [0.25 – 1.22]	0.96 (0.11) [0.77 – 1.20]	1.06 (0.05) [0.98 – 1.16]	0.08 (1.11) [-0.07 – 0.25]	0.99 (0.01) [0.97 – 1.02]

[a]GLM, gamma distribution and log link. [b]GLM, normal distribution, identity link. [c]GLM, normal distribution, log link. Clustered robust standard errors obtained. Analysis restricted to the pre-treatment period (N=180)

© 2021 Apenteng BA et al. *JAMA Network Open.*

eTable 3. Falsification Test: Patient Deduction[a] (N = 544)

	Unmatched Sample (N=544)	Matched Sample (N=360)
	exp(b)<br>(S.E)<br>[95% CI]	*exp(b)*<br>(S.E)<br>[95% CI]
Program Participation<br>(Ref: Not participating)	1.07<br>(0.06)<br>[0.95 – 1.20]	0.94<br>(0.06)<br>[0.84 – 1.06]
Time: Post program implementation (Ref: pre-program)	1.05*<br>(0.02)<br>[1.01 – 1.09]	1.05<br>(0.03)<br>[0.99 – 1.10]
Traditional DID Estimator<br>(Program Participation * Time)	0.99<br>(0.02)<br>[0.96 – 1.02]	1.00<br>(0.02)<br>[0.95 – 1.04]

*p<0.05;**p<0.01;***p<0.001

[a]GLM, normal distribution, log link. Clustered robust standard errors obtained. Analysis restricted to the pre-treatment period. Models adjusted for all hospital and market covariates listed in Table 1 and included state and year fixed effects.

© 2021 Apenteng BA et al. *JAMA Network Open.*

eTable 4. Two Part DID Model: Donations and Contributions

	Unmatched Sample (N=544)		Matched Sample (N=360)	
	b<br>(Robust S.E)<br>[95% CI]	*exp (b)*<br>[95% CI]	*b*<br>(Robust S.E)<br>[95% CI]	*exp (b)*<br>[95% CI]
**First Part**				
Program Participation<br>(Ref: Not participating)	0.04<br>(0.40)<br>[-0.75 – 0.83]	1.04<br>[0.47 – 2.29]	-0.09<br>(0.49)<br>[-1.05 – 0.87]	0.91<br>[-0.35 – 2.39]
Time: Post program implementation (Ref: pre-program)	-0.02<br>(0.23)<br>[-0.48 – 0.44]	0.98<br>[0.62 – 1.55]	0.14<br>(0.28)<br>[-0.40 – 0.68]	1.15<br>[0.67 – 1.97]
Traditional DID Estimator<br>(Program Participation * Time)	0.02<br>(0.28)<br>[-0.52 – 0.57]	1.02<br>[0.59 – 1.77]	0.09<br>(0.36)<br>[-0.61 – 0.78]	1.09<br>[0.54 – 2.18]
**Second Part**	*b*<br>(Robust S.E)	*exp (b)*<br>[95% CI]	*b*<br>(Robust S.E)	*exp (b)*<br>[95% CI]
Program Participation<br>(Ref: Not participating)	-0.48<br>(0.61)<br>[-1.67 – 0.71]	0.62<br>[0.19 – 2.03]	-1.55**<br>(0.58)<br>[-2.69 – -0.40]	4.71***<br>[0.07 – 0.67]
Time: Post program implementation (Ref: pre-program)	0.40<br>(0.30)<br>[-0.18 – 0.98]	1.49<br>[0.84 – 2.66]	0.54*<br>(0.24)<br>[-1.02 – -0.06]	1.72*<br>[0.36 – 0.94]
Traditional DID Estimator<br>(Program Participation * Time)	1.29***<br>(0.31)<br>[0.68 – 1.89]	3.63***<br>[1.97 – 6.62]	2.01***<br>(0.33)<br>[1.35 – 2.67]	7.46***<br>[3.86 – 14.4]

*p<0.05;**p<0.01;***p<0.001. First part : Probit Model; Second Part: GLM, gamma distribution and log link. Note: betas are non-exponentiated. Analysis restricted to the pre-treatment period. Models adjusted for all hospital and market covariates listed in Table 1 and included state and year fixed effects.

© 2021 Apenteng BA et al. *JAMA Network Open.*

eTable 5. Unmatched DID Estimation: Sample Excluding Hospital Stabilization Program Participants (n = 464)

	Contribution[a]	Total Margin[b]	Days Cash on Hand[c]	Debt Asset[c]	Average Plant Age[c]	FSI[b]
	exp(b)<br>(S.E)<br>[95% CI]	*b*<br>(S.E)<br>[95% CI]	*exp(b)*<br>(S.E)<br>[95% CI]	*exp(b)*<br>(S.E)<br>[95% CI]	*exp(b)*<br>(S.E)<br>[95% CI]	*b*<br>(S.E)<br>[95% CI]
Program Participation (Ref: Not participating)	0.30<br>(0.25)<br>[0.06 – 1.54]	-2.88<br>(2.26)<br>[-7.32 – 1.56]	1.96<br>(0.93)<br>[0.78 – 4.96]	1.24<br>(0.38)<br>[0.67 – 2.28]	11.7<br>(23.3)<br>[28.9 – 10405.5]	-0.02<br>(0.12)<br>[-0.23 – 0.26]
Time: Post program implementation (Ref: pre-program)	0.78<br>(0.36)<br>[0.32 – 1.92]	-2.89*<br>(1.37)<br>[-5.59 – -0.18]	0.99<br>(0.43)<br>[0.43 – 2.31]	1.28<br>(0.23)<br>[0.90 – 1.83]	0.72<br>(0.57)<br>[0.003 – 0.15]	-0.18*<br>(0.08)<br>[-0.33 - -0.02]
Traditional DID Estimator (Program Participation * Time)	23.2***<br>(16.8)<br>[5.62 – 96.0]	8.47***<br>(1.83)<br>[4.88 – 12.1]	1.25<br>(0.25)<br>[0.85 – 1.83]	1.04<br>(0.15)<br>[0.79 – 1.39]	0.90<br>(0.17)<br>[0.26 – 1.30]	0.27***<br>(0.07)<br>[0.12 – 0.41]

*p<0.05;**p<0.01;***p<0.001

[a]GLM, gamma distribution and log link. [b]GLM, normal distribution, identity link. [c]GLM, normal distribution, log link. Clustered robust standard errors obtained. Models adjusted for all hospital and market covariates listed in Table 1 and included state and year fixed effects. The analytical sample only included data from 20 out of the 22 hospitals that have ever participated in the Rural Hospital Stabilization Program. These 20 hospitals were excluded from this analysis.

© 2021 Apenteng BA et al. *JAMA Network Open.*

eTable 6. Matched DID Estimation: Sample Excluding Hospital Stabilization Program Participants (n = 312)

	Contribution[a]	Total Margin[b]	Days Cash on Hand[c]	Debt Asset[c]	Average Plant Age[c]	FSI[b]
	exp(b)<br>(S.E)<br>[95% CI]	*b*<br>(S.E)<br>[95% CI]	*exp(b)*<br>(S.E)<br>[95% CI]	*exp(b)*<br>(S.E)<br>[95% CI]	*exp(b)*<br>(S.E)<br>[95% CI]	*b*<br>(S.E)<br>[95% CI]
Program Participation (Ref: Not participating)	0.24<br>(0.24)<br>[0.04 – 1.71]	-2.38<br>(2.61)<br>[-2.74 – 7.51]	1.95<br>(0.98)<br>[0.72 – 5.21]	1.09<br>(0.53)<br>[0.42 – 2.85]	1.75<br>(0.83)<br>[0.69 – 4.45]	0.17<br>(0.15)<br>[-0.12 – 0.46]
Time: Post program implementation (Ref: pre-program)	0.62<br>(0.37)<br>[0.20 – 2.02]	-2.92*<br>(1.28)<br>[-5.44 – -0.40]	0.93<br>(0.31)<br>[0.48 – 1.78]	1.11<br>(0.52)<br>[0.44 – 2.78]	0.75*<br>(0.19)<br>[1.02– 1 76]	-0.12<br>(0.10)<br>[-0.32 - 0.07]
Traditional DID Estimator (Program Participation * Time)	15.8***<br>(11.5)<br>[3.78 – 65.7]	7.57***<br>(1.86)<br>[3.92 – 11.2]	1.32<br>(0.24)<br>[0.92 – 1.89]	0.88<br>(0.16)<br>[0.61 – 1.27]	0.75<br>(0.17)<br>[0.46 – 1.21]	0.23*<br>(0.10)<br>[0.04 – 0.42]

*p<0.05;**p<0.01;***p<0.001

[a]GLM, gamma distribution and log link. [b]GLM, normal distribution, identity link. [c]GLM, normal distribution, log link. Clustered robust standard errors obtained. Models adjusted for all hospital and market covariates listed in Table 1 and included state and year fixed effects. The analytical sample only included data from 20 out of the 22 hospitals that have ever participated in the Rural Hospital Stabilization Program. These 20 hospitals were excluded from this analysis.

© 2021 Apenteng BA et al. *JAMA Network Open.*

eFigure 1. Bias Reduction Following Matching

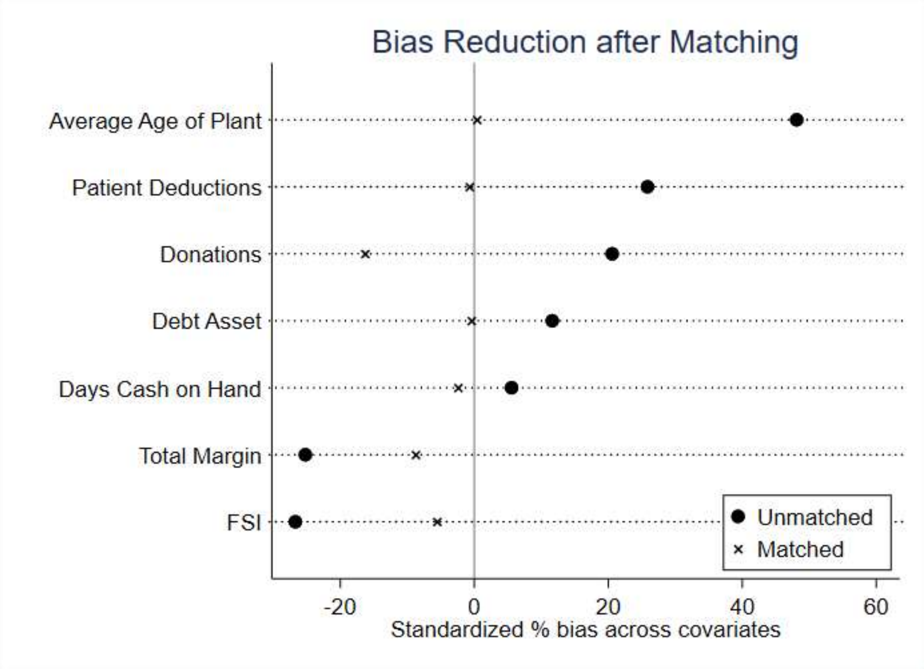

© 2021 Apenteng BA et al. *JAMA Network Open.*

eFigure 2. Unadjusted Linear Trends in Patient Deductions

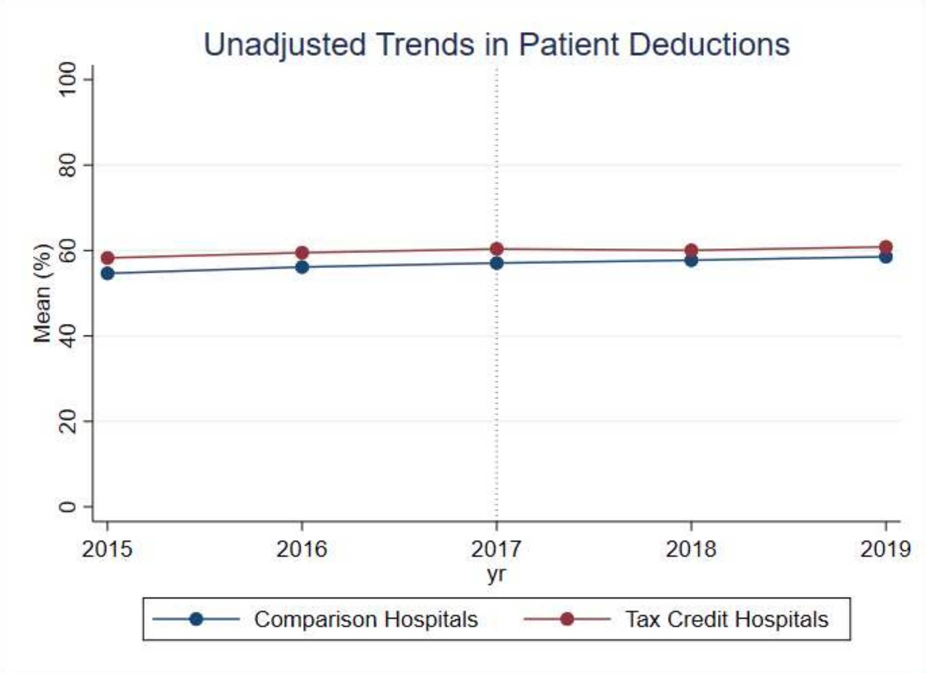

© 2021 Apenteng BA et al. *JAMA Network Open.*

eFigure 3. Event Study Graphs of DID Estimates: Patient Deductions

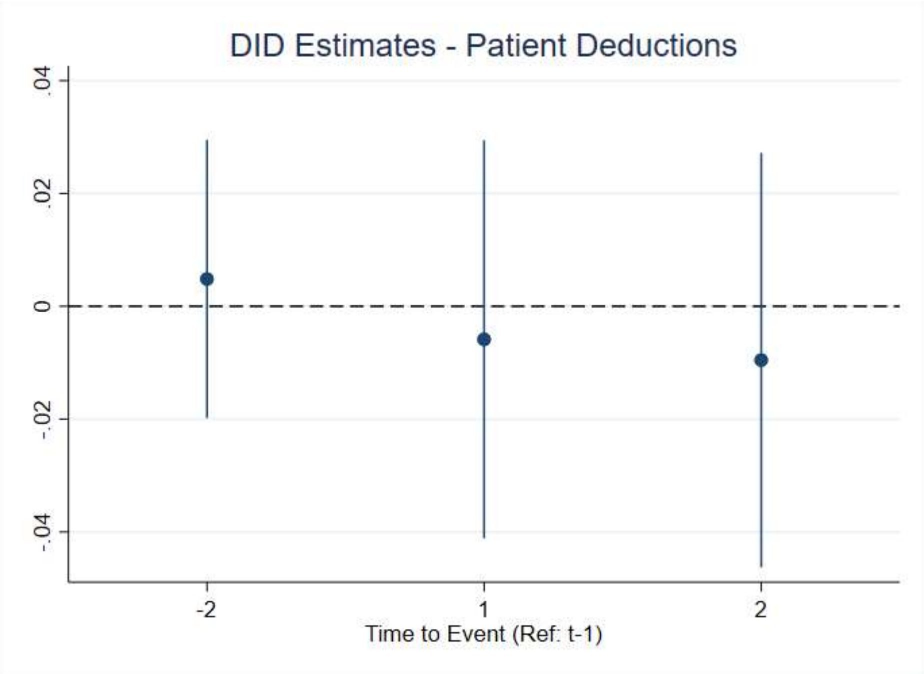

© 2021 Apenteng BA et al. *JAMA Network Open.*
